# 62541 Continuity of Care for Patients with Chronic Gastrointestinal Disease: A Latent Class Analysis of Patients With High-Intensity Specialty Care Needs

**DOI:** 10.1017/cts.2021.713

**Published:** 2021-03-30

**Authors:** Shirley Cohen-Mekelburg, Liberty Greene, Akbar Waljee, Tim Hofer, Sameer Saini, Donna Zulman

**Affiliations:** 1 University of Michigan; 2 VA Ann Arbor; 3 Stanford University

## Abstract

ABSTRACT IMPACT: Grouping patients with potentially high intensity specialty care needs based on their propensity for healthcare continuity patterns can inform the development of personalized care coordination and care navigation interventions OBJECTIVES/GOALS: To examine variation in healthcare continuity patterns across primary care, mental health care, and specialty care for a patient population with chronic gastrointestinal conditions and a high risk for healthcare utilization. METHODS/STUDY POPULATION: We analyzed data for Veterans Affairs patients with chronic gastrointestinal disease (cirrhosis, inflammatory bowel disease, chronic pancreatitis) whose 1-year hospitalization risk was ≥90th percentile in 2014, and who had a minimum of 4 office visits. To assess continuity, we examined frequency of office visits, number of outpatient providers, and two established continuity of care measures (the usual provider of care index and the Bice-Boxerman continuity of care index) over 12 months. We used latent class analysis to categorize patients into classes based on overall, primary care (PCP)-specific, gastroenterology (GI)-specific, and mental health specific-healthcare continuity patterns. RESULTS/ANTICIPATED RESULTS: The 26,751 Veterans in the analytic cohort had a mean of 13.3 (sd 8.63) office visits and 7.2 (sd 3.83) providers per patient. Patients were classified into five phenotypes: (1) moderate overall use and continuity; (2) low overall continuity of care; (3) high GI- and PCP-specific continuity of care; (4) low overall continuity of care with some mental health use; and (5) high utilization with substantial mental health use. In the subsequent year, 11,259 (42.1%) patients had a hospitalization and 16,167 (60.4%) had an emergency department visit. These groups varied in their sociodemographic and clinical characteristics, and in their risk for hospitalization and emergency department use. DISCUSSION/SIGNIFICANCE OF FINDINGS: Latent class analysis revealed five distinct primary and specialty care utilization patterns. Grouping patients with high intensity specialty care needs based on their propensity for healthcare continuity patterns can inform the development of personalized care navigation interventions.

